# A Rare Case of Nasal Chondromesenchymal Hamartoma Presenting With Respiratory Distress in a Newborn: A Case Report

**DOI:** 10.1155/crot/1090975

**Published:** 2026-01-28

**Authors:** Mikiyas Olani, Mohammed Juhar, Siraw Girum, Amanuel Damie, Alemwork Amare, Abel Daniel

**Affiliations:** ^1^ Department of Otolaryngology-Head and Neck Surgery, College of Health Sciences, Addis Ababa University, Addis Ababa, Ethiopia, aau.edu.et; ^2^ Department of Pathology, College of Health Sciences, Addis Ababa University, Addis Ababa, Ethiopia, aau.edu.et; ^3^ Department of General Surgery, College of Health Sciences, Addis Ababa University, Addis Ababa, Ethiopia, aau.edu.et

**Keywords:** benign nasal mass, case report, chondromesenchymal hamartoma, nasal obstruction, neonatal nasal mass

## Abstract

**Introduction:**

Nasal chondromesenchymal hamartoma (NCMH) is a rare cause of nasal mass in infants and children. It was first described in 1998, and since then, only 63 previous cases have been reported.

**Case Report:**

Here, we report a case of a 4‐day‐old neonate with a right‐sided nasal mass presenting with respiratory distress since birth. MRI was suggestive of chondromesenchymal hamartoma, for which endoscopic excision was done with complete removal of the tumor. The patient was diagnosed and successfully managed in our setup.

**Discussion:**

NCMH is a rare cause of nasal obstruction in neonates, with a similar clinical presentation to other known nasal masses. Physical examination, imaging, histopathology, and molecular tests are combined to diagnose such cases. The curative management currently recommended is surgery.

**Conclusion:**

It is always prudent to consider all possible differentials in neonates presenting with a nasal mass. Our report focuses on the role of proper examination, imaging techniques, and histologic evaluation for proper diagnosis and follow‐up in a resource‐limited setup.

## 1. Introduction

Congenital nasal masses are rare, with an incidence of 1 in 20,000–40,000, and the most common among these are nasal gliomas, encephaloceles, and dermoid cysts [1, 2]. They are relatively rare causes for respiratory distress in newborns and hence present a diagnostic challenge for practitioners, especially in a resource limited setup [3]. Nasal chondromesenchymal hamartoma (NCMH) is a benign tumor closely resembling the chest‐wall hamartomas of infancy in histologic appearance. It is currently understood to be one among Digital Information Center for Environment Research (DICER1)‐associated neoplasms [4]. The DICER1 gene codes for the dicer protein, which is responsible for microRNA production, which in turn is responsible for the expression of more than 30% of protein coding genes. A mutation in the DICER1 gene leads to DICER‐associated neoplasms, one among which is NCMH [4]. The first description of NCMH was reported by McDermott et al. as a series of nasal masses that occur in children, predominantly in infants less than 3 months of age [5]. According to the most recent systematic review so far and one other report, there have been only about 63 cases reported worldwide. The mean age of presentation is 5.1 years with most cases presenting in under 1 year of age. About 15 adult cases of NCMH have been reported in the published literature so far [6, 7]. It is a rare pathology among neonatal nasal masses [3, 8]. To the best of our knowledge, only one case is reported from Africa, and we are reporting the second case so far [6, 9]. Considering the rarity of the condition and the challenges in diagnosis, management, and follow‐up of these cases, we believe it is important to report every case. Here, we report a newborn who was diagnosed with NCMH.

## 2. Case Report

A 4‐day‐old female neonate born to a Para 8 mother (all alive) after 9 months of amenorrhea. Labor started spontaneously, and delivery was at home due to the inconvenience of reaching the health center. At the age of 5 h, she was taken to the local health center, which referred her to the local hospital. Upon evaluation, she was having respiratory distress with fast breathing and cyanosis, which improved with crying. There was difficulty passing the nasogastric tube number 6 French through the bilateral nasal cavity. Oral airway was secured, and the patient was put on facemask oxygen support. Otherwise, the baby has no history of loss of consciousness, abnormal body movement, or excessive secretions. The mother has no history of drug intake during pregnancy and no history of smoking or alcohol intake and was supplemented with folate and iron during antenatal care follow‐up.

With the possible consideration of bilateral nasal obstruction, he was referred to our hospital for better management. Upon physical examination, the patient has signs of distress with fast breathing and chest in drawing with open mouth breathing. Pulse rate was 180 regular and full in volume. Respiratory rate ranged from 65 to 70 breaths per minute, and oxygen saturation was 87% on oral airway and reached 99% with facemask oxygen support. Axillary temperature was 37.9°C and capillary refill was less than 2 s. On nasal examination, application of cotton shows no airflow. Oral airway was in situ and functional, with an orogastric tube also in place. The chest examination showed severe subcostal and intercostal retraction with bilateral rhonchi in the lower two‐thirds of the lung fields. The neurologic examination showed an alert and normotonic neonate with normal Moro reflexes.

A pinkish soft tissue mass was visualized in the right nostril (Figure 1). Nasal examination with a 4 mm nasal flexible endoscope was tried, and it was difficult to pass in both nostrils; an enhanced paranasal sinus computed tomography (CT) scan was sent.

**Figure FIGURE 1:**
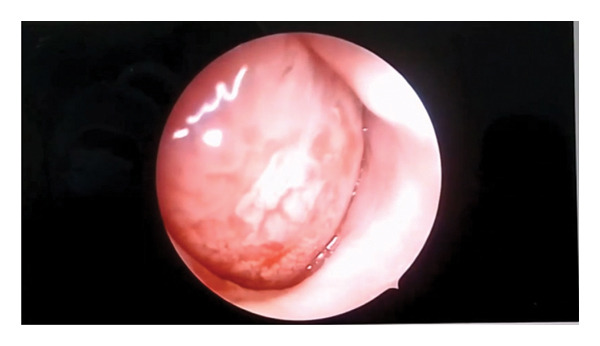
Endoscopic view of a pinkish globular mass filling the right side of the nasal cavity.

CT scanning was done. The finding was suggestive of a large right side nasal cavity mass measuring 2.6 by 2.4 by 3.5 cm with an enhancing solid and cystic component, with complete obstruction of the right nasal cavity and compression of the medial wall of the right maxillary sinus. The nasal septum pushed and narrowed the left nostril. The mass has no clear plane at the ethmoidal skull base. We could not retrieve the CT scan image file to be presented here, as it was lost from our database. Magnetic resonance imaging (MRI) finding was a relatively well‐defined right sinonasal lesion involving the maxillary and ethmoid sinus region, which is predominantly T1 isointense to the muscle with peripheral areas of hyperintensity and T2/FLAIR heterogeneously hyperintense to the muscle and cortex (Figures 2(a) and 2(b)). It has pushed the turbinate and the nasal septum to the left side. The medial wall of the right orbit is pushed laterally by the lesion. It shows no clear sign of invasion of adjacent structures or extension to the orbits or intracranial cavity. There was intense inhomogeneous postcontrast enhancement. Based on the imaging findings, a diagnosis of NCMH was made.

Figure FIGURE 2(a) Coronal section T2 MRI showing a heterogeneous mass with the typical cystic enhancement on T2 with a hypodense solid area. (b) Sagittal section of a T2 MRI showing the clear delineation from the intracranial space and a well‐delineated mass with solid and cystic components.

**Figure:**
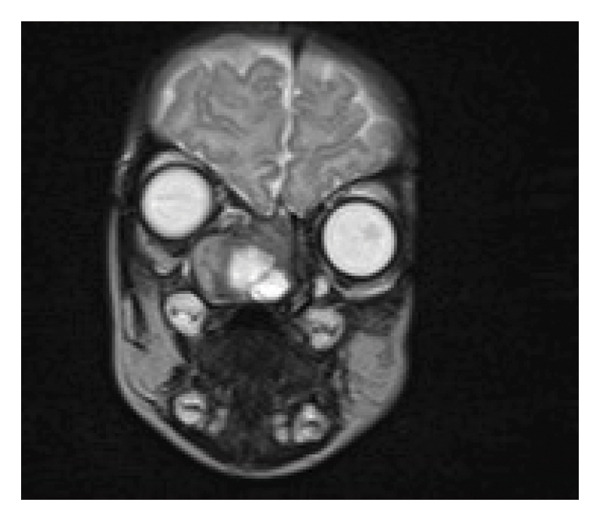
(a)

**Figure:**
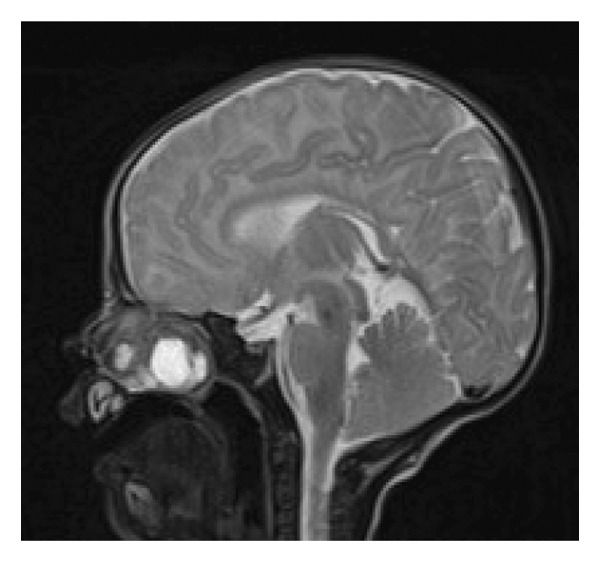
(b)

On baseline echocardiography, there was a small patent foramen ovale (2 mm) shunting left to right with no other cardiac anomaly. Screening abdominopelvic ultrasound was done and was unremarkable. Preoperative biopsy was not done, considering the benign nature of the mass.

With the assessment of nasal cavity mass secondary to NCMH, it was planned to do elective surgery, but the patient developed a hospital‐acquired infection with pyogenic meningitis and was on treatment, which delayed the surgical intervention.

Surgery was done at the age of 41 days with an endoscopic approach. A zero‐degree 4‐mm endoscope was advanced, and the nasal cavity was examined. There was a pinkish mass filling the right nasal cavity, pushing the septum to the left. The mass was debulked in a piecemeal fashion till its attachment at the anterior ethmoidal skull base, and the attachment was thoroughly cauterized. Grossly, the mass was composed of a solid and cystic component with some intervening cartilage; it was difficult to send for margin assessment since it was removed in a piecemeal fashion (Figure 3). Histologic examination of the specimen showed the presence of mature cartilage with a myxoid stroma and the presence of spindle cells (Figure 4). Although immunohistochemistry and molecular tests for DICER1 status were planned, it was not done because of the unavailability in out setup.

**Figure FIGURE 3:**
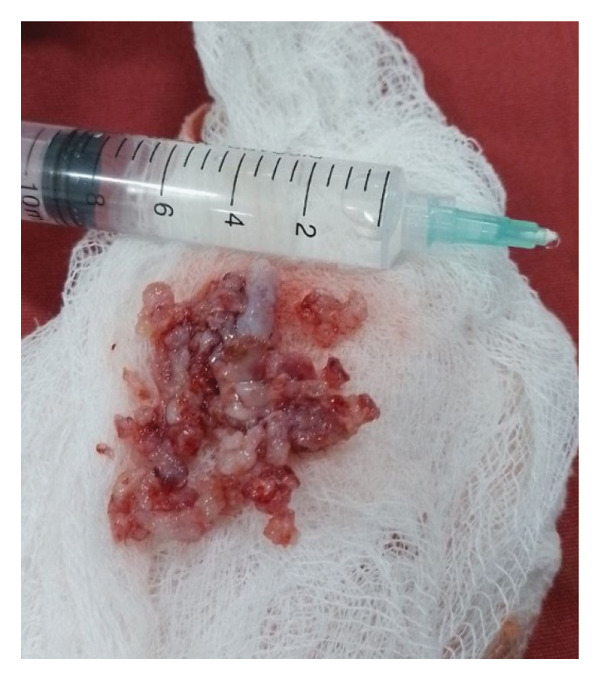
Intraoperative picture depicting the removed tumor in piecemeal, having cartilaginous and soft tissue components.

**Figure FIGURE 4:**
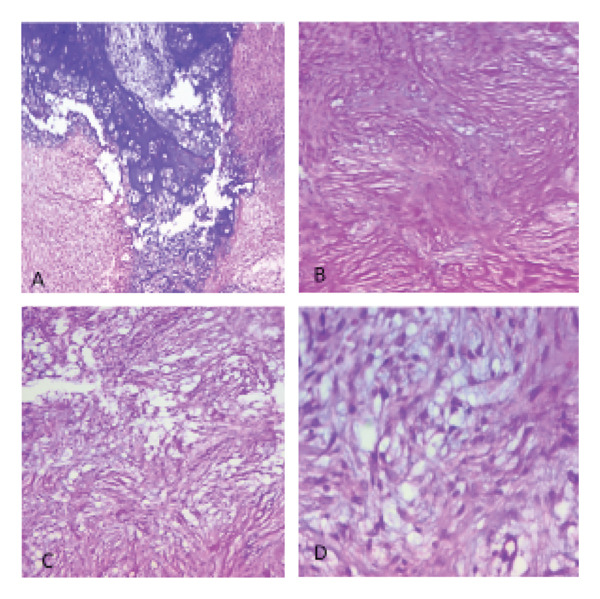
H&E: (A) × 40‐demarcated lobules of mature cartilage, (B) × 100‐collagen fibers, (C) × 100‐myxoid stroma, and (D) × 400‐Bland spindle cells set in myxoid stroma.

The postoperative course of the patient was uneventful, and the patient was discharged home on the third day. At 1 week, endoscopic examination was done, which was unremarkable with well‐healing mucosa. The patient was feeding well with no airway problems.

## 3. Discussion

The term chondromesechymal hamartoma (CMH) was first used by McDermott et al. in a paper published in 1998 [5]. His original report included 7 children, 6 of whom were less than 3 months at the time of diagnosis. He further described that their clinical presentation in infancy and cartilaginous nature resembles the mesenchymal hamartoma of the chest wall [5]. Recent studies have found a link between NCMH and DICER1, a familial tumor predisposition gene [4].

CMH generally tends to occur more frequently in infants and younger children although 16 cases of adult‐onset CMH have been reported so far, including 15 cases in the most recent systematic review, and another case reported in a separate publication by Askari et al. [6, 7]. The incidence is twice as high in males as in females. The nasal cavity is the second most frequent site for occurrence, following the paranasal sinuses [6]. Our patient is a male neonate, 4 days old at diagnosis, and mass was located in the right nasal cavity attaching at the ethmoidal skull base.

The commonest clinical presentation of patients includes nasal obstruction, nasal mass, orbital signs, respiratory distress, and facial swelling. Symptom depends on the extent of the mass, with some tumors extending to the intracranial space and the orbit presenting with neurologic symptoms like hydrocephalus and orbital signs like proptosis and limitation of eye movement [10]. Our patient presented with cyanotic episodes with respiratory distress, which improved with crying, and had a visible mass in the right nasal cavity at presentation.

Imaging plays a crucial part in differentiating NCMH from other masses. The differential diagnosis based on imaging includes nasal glioma, inverted papilloma, giant cell reparative granuloma, ossifying fibroma, and aneurysmal bone cyst [10]. In our case, the mass had no intracranial communication and no calcified component, which rules out the first two differentials. Inverted papilloma tends to occur in the 50–60 age range [11]. Aneurismal bone cysts are even rarer in infancy [12]. Malignancies which mimic NCMH include rhabdomyosarcoma, esthesioneuroblastoma, and chondrosarcoma. Except for rhabdomyosarcoma, the other malignancies are usually seen only in adolescents [10].

In a series of 15 cases of NCMH by Johnson et al., the findings on CT scan include the presence of orbital involvement, paranasal sinus extension, bony remodeling, enhancement, intracranial extension, internal calcification, and cystic component with descending order of presence [10]. An MRI study is better in evaluating the soft tissue characterization and extension into adjacent structures like the orbit and intracranial space. MRI features mimic the histologic composition of the tumor with T2 hyperintensity of the cystic and myxoid component and distinct T2 hypointensity of the mineralized and fibrosed component [13]. In our case, the distinct CT scan and MRI features led us to consider NCMH.

Gross description of NCMH is soft to firm mass and pink to tan in color. Histologic composition of the tumor is characterized by the presence of mature and immature cartilage surrounded by myxoid stroma, fibro‐osseous, and mesenchymal components. There are associated chondrocytes and spindle cells with very low mitotic activity and no features of atypia [9]. The differential diagnosis based on histology includes nasal polyps, inverted papilloma, or fibrocartilagenous mass [6]. So far, only one case of malignant transformation of NCMH has been reported with areas of cellular atypia with mitosis and necrosis present histologically [14]. Almost all cases of CMH stain positive for SMA, S100, and vimentin on immunohistochemistry [7, 12]. In our case, the pathologic diagnosis relied on microscopy with the presence of a mature cartilage island in myxoid stroma and the presence of spindle cells. Immunohistochemical analysis was not available in our setup, and the patient’s family could not afford to do it outside.

Underlying genetic predisposition is thought to be a risk factor for the development of NCMH, and hence, the predominantly affected age group is the very young [15]. DICER1 mutation is associated with NCMH occurrence, and in one series of 8 patients, 6 had a germline mutation in DICER1 [15]. Other tumors with DICER1 association include pleuropulmonary blastoma, ovarian sex cord–stromal tumors, juvenile granulosa cell tumor, Sertoli–Leydig cell tumor, and gynandroblastomas [4, 6, 15]. NCMH can present earlier than other tumors because of its location [6, 9, 13]. In our patient, a physical examination and abdominopelvic ultrasound were done, but no other tumors were detected. It is generally recommended to send patients diagnosed with NCMH for genetic testing to look for DICER1 mutation [16]. Genetic screening was not done as it was not available in our country and patients could not afford to do it abroad. Despite this, we would like to iterate that rare disorders like NCMH can be diagnosed and managed even in resource limited setups if we have a keen look into on the clinical picture and imaging findings whenever there is a lack of advanced confirmatory tests like immunohistochemistry or genetic testing.

The management of NCMH is primarily surgical, which is curative once complete excision is undertaken. The location and extent of the tumor might make the resection difficult, resulting in residual tumor and recurrence in some cases. Surgical approach could be endoscopic or open, and tumors limited to the nasal cavity can be successfully removed with an endoscopic approach [8, 17, 18]. Open neurosurgical approaches could be undertaken in cases with significant intracranial extension [7]. In our case, we were able to successfully remove the tumor via transnasal endoscopic approach. The tumor base was cauterized with bipolar cautery after gross total tumor removal was achieved. No additional treatment was provided. The patient was followed up at the first week postsurgery with endoscopy. There was well‐healing mucosa and no residual disease.

It is strongly recommended to have a follow‐up postsurgery, as there have been reports of malignant transformation and persistent or recurrent disease. The patient can also go on to develop other syndromic manifestations of the DICER tumors. In the reported literature so far, the follow‐up period averages at 24 months [4, 6]. To our knowledge, there is no guideline recommendation for the duration of follow‐up for patients with NCMH. Askari et al. had a follow‐up of their patient with endoscopy every 2 months for the first year, then every 4 months for 5 years, and then every 6 month after that. They also suggested doing follow‐up CT scan if patient has signs of nasal obstruction which lasts more than 2 weeks [7]. Our patient was followed at 12 months postsurgery, and there was no sign of recurrence or residual disease. Further follow‐up was not possible since patient was lost to follow‐up.

## 4. Conclusion

When a neonate presents with a nasal mass, it is prudent to consider all possible differential diagnoses, including rare tumors like NCMH. Clinical presentation can mimic other differential diagnoses with the presence of nasal mass, respiratory distress, and orbital and neurologic symptoms. Careful examination of MRI and CT scan findings can be used to better characterize tumor composition and extent, which will aid in diagnosis and surgical planning. Diagnosis can be supported with histologic examination by the presence of mixed mesenchymal elements like cartilage islands, myxoid stroma, and mesenchymal cells with no atypical features. It is prudent to do screening for DICER1 mutation for every case of NCMH if possible. Surgery is the only successful mode of management, with complete excision resulting in a cure. Long‐term follow‐up is mandatory as patients may manifest with subsequent tumors in the DICER1 syndrome.

## Author Contributions

**Dr Mikiyas Olani**: conceptualization (lead), writing–original draft (lead), and writing–review and editing (equal); **Dr Mohammed Juhar**: writing–review and editing (equal); **Dr Alemwork Amare**: conceptualization (equal) and writing–original draft (equal); **Dr Siraw Girum**: writing–review and editing (equal); **Dr Amanuel Damie**: writing–original draft (supporting) and writing–review and editing (equal); **Dr Abel Daniel**: conceptualization (equal) and writing–original draft (supporting).

## Funding

The authors received no financial support for the research, authorship, or publication of this article.

## Consent

Informed written consent has been obtained for publication and can be presented upon request.

## Conflicts of Interest

The authors declare no conflicts of interest.

## Data Availability

Data used to support the findings of this study are available from the corresponding author upon reasonable request.
